# Plants under Stress: Involvement of Auxin and Cytokinin

**DOI:** 10.3390/ijms18071427

**Published:** 2017-07-04

**Authors:** Agnieszka Bielach, Monika Hrtyan, Vanesa B. Tognetti

**Affiliations:** Mendel Centre for Plant Genomics and Proteomics, Central European Institute of Technology (CEITEC), Masaryk University, Kamenice 5, Czech 62500, Brno, Czech Republic; agnieszka.bielach@ceitec.muni.cz (A.B.); monika.hrtyan@ceitec.muni.cz (M.H.)

**Keywords:** auxin, cytokinin, abiotic stress, crosstalk, growth, adaptation, ROS

## Abstract

Plant growth and development are critically influenced by unpredictable abiotic factors. To survive fluctuating changes in their environments, plants have had to develop robust adaptive mechanisms. The dynamic and complementary actions of the auxin and cytokinin pathways regulate a plethora of developmental processes, and their ability to crosstalk makes them ideal candidates for mediating stress-adaptation responses. Other crucial signaling molecules responsible for the tremendous plasticity observed in plant morphology and in response to abiotic stress are reactive oxygen species (ROS). Proper temporal and spatial distribution of ROS and hormone gradients is crucial for plant survival in response to unfavorable environments. In this regard, the convergence of ROS with phytohormone pathways acts as an integrator of external and developmental signals into systemic responses organized to adapt plants to their environments. Auxin and cytokinin signaling pathways have been studied extensively. Nevertheless, we do not yet understand the impact on plant stress tolerance of the sophisticated crosstalk between the two hormones. Here, we review current knowledge on the function of auxin and cytokinin in redirecting growth induced by abiotic stress in order to deduce their potential points of crosstalk.

## 1. Introduction

More than half a century has elapsed since the discovery that the phytohormones auxin and cytokinin are required to induce cell division and growth in plant tissue culture [1]. Since then, the two phytohormones have been recognized as the main regulators of plant development [2,3,4,5,6,7,8,9,10,11]. It is not surprising, therefore, that their biology has been studied extensively and that the underlying mechanisms of their action have captivated several generations of scientists. These two hormones have specific biosynthetic, homeostasis, transport and signal transduction pathways, which by now are well described. The literature on these topics is so comprehensive that it is impossible to encompass the information on auxin and cytokinin biology in this review [12,13,14,15,16,17,18]. In recent years, auxin and cytokinin crosstalk has been studied extensively at all levels (synthesis, perception and transport), and we are beginning to understand how these networks interact to control a wide variety of plant responses [3,8,19,20,21,22].

As sessile organisms, plants are continuously exposed to an enormous amount of external stimulus. Such external stimuli include, for example, a wide and diverse range of extreme weather conditions, which may appear unpredictably. Being aerobic organisms, plants use reactive oxygen species as a signaling molecule. However, if the accumulation of reactive oxygen species (ROS) occurring under suboptimal growth conditions, such as abiotic stresses, reaches toxic levels, their oxidative effects on plant cells can be lethal. Therefore, plants have developed mechanisms enabling quick recognition, distinction and response during a stressful encounter by, on the one hand, maintaining ROS levels at nontoxic concentrations through a complex battery of antioxidant systems and, on the other hand, by ROS interacting with phytohormone pathways [23,24]. To survive and adapt to new conditions, many plants redirect their growth through various morphological, physiological and biochemical responses that decrease stress exposure, limit damage or facilitate the repair of damaged systems. In other words, plants have managed to acquire an escape or survival strategy to deal with stress by modifying some of their morphological and physiological traits. In particular, cooperative action of the two phytohormones auxin and cytokinin with stress-induced ROS signals connects plant development with plant responses to environmental changes [25,26,27,28,29,30,31]. The resulting crosstalk between ROS and auxin and cytokinin allows plants to adjust their development and growth to unfavorable external cues. In this review, we focus on the roles of auxin and cytokinin in plant growth and development while emphasizing two major areas: (i) how plants maintain growth via balancing both hormonal pathways along with ROS signals; and (ii) inferring potential stress-auxin-cytokinin crosstalk regulatory hubs that might act as pivotal junctions between the hormone orientation and stress-induced plant growth. 

## 2. Role of Auxin and Cytokinin during Plant Response to Abiotic Stress

Physiological and genetic studies have dissected the functions of individual hormonal pathways in different developmental contexts, and recent advances have shown that hormones act through a network of interacting responses rather than through isolated linear pathways [22]. Here, we focus on the effect of abiotic stress on the control points of auxin and cytokinin pathways, which could explain in part the stress-induced changes in plant architecture and growth patterns. We summarize how abiotic stress influences auxin and cytokinin concentrations, transport and responses. At the same time, we would like to draw attention to important checkpoints for approaches in modern biotechnology aiming to improve plant yields under stress conditions. The genes from cytokinin and auxin pathways studied to date that respond to abiotic stress, as well as their respective roles in stress-tolerance are summarized in Supplementary Table S1. 

### 2.1. Targets of Auxin and Cytokinin Biosynthesis Modulated by Stress

The youngest leaves containing the largest amounts of auxin exhibit the greatest capacity for de novo auxin synthesis [32] and usually exhibit increased stress avoidance [33]. Recent findings suggest that a functional YUCCA (YUC) pathway of auxin biosynthesis may be exploited to alter plant responses to the environment [34,35,36]. In *Arabidopsis thaliana* and barley (*Hordeum vulgare*), for example, inhibition of the expression of YUC2 and YUC6 genes by high temperatures leads to a local decrease in endogenous auxin levels in developing anthers, which in turn results in male sterility [37,38]. Exogenous application of indole-3-acetic acid (IAA) efficiently rescues male sterility in both species [38]. This suggests that tissue-specific decrease in auxin is the primary response to such environmental change as an increase in temperature, which leads to the abortion of pollen development and, as a result, a decrease in yield. Additionally, *Arabidopsis* plants overexpressing *YUC6* or transgenic poplar expressing *Arabidopsis YUC6* under the control of the oxidative stress-inducible *SWPA2* promoter display enhanced IAA-related phenotypes and exhibit improved drought and oxidative stress resistance [39,40]. Recent studies show that besides harboring a flavin-containing monooxygenase (FMO) domain involved in IAA biosynthesis, *YUC6* contains FAD- and NADPH-dependent thiol-reductase activity (TR) domains overlapping with the FMO domain. Interestingly, the stress-related phenotypes of YUC6 overexpressors do not appear to be linked to IAA overproduction, but rather to TR activity of YUC6 [39]. Moreover, the auxin level increased by constitutive overexpression of the *Arabidopsis* YUC6 gene in potato (*Solanum tuberosum*) [34,36] or overexpression of the *TRYPTOPHAN-2-MONOOXYGENASE* known as *iaaM* in *Arabidopsis* enhances drought tolerance [41]. Drought-resistant phenotype was correlated with auxin-dependent decrease in H_2_O_2_ and O_2−_ [34,36,41]. Elevated auxin positively modulates the expression levels of multiple abiotic stress-related genes (*RAB18*, *RD22*, *RD29A*, *RD29B*, *DREB2A* and *DREB2B*) and positively affects antioxidant enzyme activities. Interestingly, drought-resistant phenotype, reduction in ROS accumulation, induction of stress-related genes and activation of antioxidant response can be mimicked by exogenous application of IAA. In contrast, lower levels of auxin in *yuc1 yuc2 yuc6* triple mutants lead to increased ROS accumulation and decreased drought tolerance [41]. 

It is known that drought resistance is closely related to the root-to-shoot ratio of biomass [42,43]. The *YUC7* gene is induced by drought primarily in the roots, and elevated levels of free auxin in *Arabidopsis* activation-tagged mutant *yuc7-1D* promotes root growth and enhances root architecture. Accordingly, *yuc7-1D* plants are resistant to drought and show upregulation of drought-responsive genes [35]. Mutation in the rice (*Oryza sativa*) *CONSTITUTIVELY WILTED1* (*COW1*) gene, which encodes a new member of the YUC protein family, leads to a lower root-to-shoot ratio of biomass and, as a consequence, unbalanced water homeostasis [42].

Active cytokinins are adenine derivatives with isoprenoid or an aromatic side chain attached to the *N*-6-position of the adenine ring. The most common group present in plants has an isoprenoid side chain and includes isopentenyladenine (iP)-, trans-zeatin (tZ)-, cis-zeatin (cZ)- and dihydrozeatin-type derivatives [44]. Less abundant are aromatic cytokinins, such as N6-(meta-hydroxybenzyl) adenine [45]. The rate-limiting step of cytokinin biosynthesis is the production of the cytokinin precursor isopentenyladenine catalyzed by ATP/ADP isopentenyltransferases (IPT). Subsequently, cytochrome P450 monooxygenases CYP735A1 and CYP735A2 catalyze the hydroxylation of isopentenyladenine-type cytokinins. Moreover, enzymes from the LONELY GUY (LOG) family convert N6-modified adenosine monophosphate derivatives into active cytokinins. Finally, cytokinins undergo irreversible degradation by oxidative side chain cleavage catalyzed by CYTOKININ OXIDASE/DEHYDROGENASE (CKX) enzymes [46]. Recent findings on cytokinin biosynthesis and signaling help to fill important gaps in the understanding of how environmental cues interact with these components to modulate plant growth development and physiology. Cytokinin function has been linked to a variety of abiotic stresses [47,48]. A decline in endogenous cytokinin levels in reaction to stress has long been observed [49,50]. In xylem sap, drought stress reduces the levels of trans-zeatin, zeatin riboside, isopentenyl adenine and isopentenyl adenosine [51]. Additionally, the transport of trans-zeatin riboside decreases drastically [52,53]. The same treatment, however, increases the amount of cytokinin 6-benzylaminopurine, suggesting that benzylaminopurine might play a role in response to stress by delaying stress-induced leaf senescence [54]. Additionally, the high concentration of benzylaminopurine induces accumulation of the osmolyte proline [55].

Participation by cytokinin biosynthesis and degradation enzymes in response to stress is likely to be dependent on their spatial and temporal expression patterns. Genetic studies in which the endogenous cytokinin levels in *Arabidopsis* were modified, either by loss of expression of *IPT* genes or by overexpression of CKX-encoding genes, show that cytokinin plays a negative role in response to stress [43,56,57,58,59,60]. Despite their being detrimental for shoot growth, analysis of *Arabidopsis CKX* overexpressor lines (*35S:CKX1*, *35S:CKX2*, *35S:CKX3*, *35S:CKX4*) and of *ipt1 ipt3 ipt5 ipt7* mutants has revealed that a reduction in cytokinin levels improves drought and salt stress tolerance [58,61]. The use of root-specific promoters prevents drawback on shoot growth. For example, targeting AtCKX1 overexpression only in roots in *Arabidopsis* and tobacco (*Nicotiana tabacum*) results in plants with larger root systems, but has no influence on shoot growth and development. Similarly, those plants exhibit higher tolerance to drought and heat stress, associated in part with the improvement in root traits [43,57]. The role of cytokinin subcellular compartmentation in roots was recently studied in transgenic barley plants overexpressing the *AtCKX1* gene targeted to various subcellular compartments and under the control of the mild, root-specific β-glucosidase promoter from maize (*Zea mays*) [59]. While the assumed cytosolic and vacuolar targeting of AtCKX1 had a negligible effect on shoot growth, secretion of the AtCKX1 protein to the apoplast had a negative effect on the development of the aerial part and on yield. Upon application of severe drought stress, all transgenic genotypes maintained a higher water potential and showed better growth and yield parameters during revitalization. The higher tolerance to drought stress exhibited by all transgenic barley plants was deliberately caused by the altered root morphology that resulted in better dehydration avoidance [59]. Therefore, morphological variations caused by altered cytokinin levels in roots or shoots play a decisive role in plant stress performance. These data highlight the importance of locally informative cytokinin signals generated by localized production and/or degradation of cytokinin for morphological changes to create stress avoidance and tolerance. 

The relevance of cytokinin level fluctuations throughout stress episodes, as well as of tissue type where such changes occur has been demonstrated by employing inducible promoters. The importance of inducing cytokinin production specifically during the onset of water stress to increase plant stress performance was confirmed by driving the expression of the *Agrobacterium tumefaciens IPT* gene by the leaf-specific drought and maturation responsive promoter of the SENESCENCE ASSOCIATED RECEPTOR PROTEIN KINASE (SARK). This strategy (*SARK::IPT*) has been used with tobacco [62], rice [63], peanut (*Arachis hypogaea*) [64], cotton (*Gossypium*) [65] and maize plants [66]. SARK promoter-regulated expression of the *IPT* gene suppresses drought-induced leaf senescence by allowing plants to produce cytokinin in tissues sensing water stress [67]. Remarkably, transgenic tobacco *SARK::IPT* plants exhibited outstanding drought tolerance and vigorous growth after a long drought period that killed the control plants [67]. Similarly, water-stressed transgenic maize *SARK::IPT* plants maintained a normal photosynthetic rate and stomatal conductance. Moreover, transgenic plants recorded only a minor decrease in the number of grains per plant, individual grain weight and plant grain yield as compared to well-watered plants [66]. Improved drought tolerance in creeping bentgrass (*Agrostis stolonifera*) expressing senescence-associate promoter *SAG12:IPT* constructs leads to an increase in endogenous iP and Z-riboside content in leaves and roots and positively effects osmotic adjustment, efficient water use and photosynthesis maintenance [68]. In these plants, high levels of cytokinin promote ROS scavenging through antioxidant accumulation and activation of antioxidant enzymes in roots and shoots. Moreover, the increment of cytokinin synthesis in the root meristem was proven to be a good tool to alleviate drought inhibition of root growth [69]. On the other hand, a negative effect on stress adaptation was observed by ectopic overproduction of endogenous cytokinin. *Arabidopsis* estradiol-inducible *IPT8* transgenic seedlings, in which estradiol and salt stress treatments were performed simultaneously, were more sensitive to stress, as indicated by reduced survival rates and chlorophyll content [70]. In tomato (*Solanum lycopersicum*), however, the constitutive endogenous expression of *SlIPT3* led to a stress-adapted phenotype [71], which is characterized by a more compact rosette size and increased shoot branching [72]. Moreover, increased cytokinin levels in young and old leaves caused several other changes that affected nutrient accumulation and photosynthesis maintenance and led to enhanced performance against salt stress. *SlIPT3* is highly expressed in old leaves and roots. In response to salt stress, its transcription is strongly repressed in tomato roots and significantly induced in young and old leaves [71]. These data suggest that increasing the cytokinin content in shoots before the onset of stress acts as a pre-adapted factor, triggering the necessary morphological alterations to prevent the negative effects of stress on plant physiology. 

The importance of tissue-specific cytokinin metabolism for adaptation against stress is evidenced by comparing the activity levels of cytokinin biosynthesis enzymes between resistant and susceptible crop varieties. In rice, for example, cytokinin metabolism is particularly relevant for panicle differentiation and grain yield, and it is known that *LOG* and *CYP735A* genes’ expression is altered by various abiotic stress conditions [73,74]. Interestingly, LOG, IPT and CYP735A enzyme activities in panicles are similar in heat-susceptible rice varieties and in heat-tolerant variety SY63 at high temperature stress. However, the stress-induced increment of total CKX activity is specific to the susceptible varieties. Moreover, reduction of cytokinin transport from root to shoot via xylem sap and decreased LOG and IPT activities seem to be responsible for the lower number of spikelets per panicle obtained under high temperature treatments in the susceptible lines [75]. Similarly, increased CKX activity was observed under high temperature stress in tobacco plants [56]. These results suggest that cytokinin catabolism is involved in high temperature stress adaptation in rice and tobacco plants. Therefore, modulation of CKXs activity represents an interesting genetic tool to increase plant yield under stress. Consistent with this assumption, knockdown rice mutants for the inflorescence meristem specific *OsCKX2* gene lead to increased primary and secondary panicle branching and growth. The effect is associated with increased tZ, iP, kinetin and dihydrozeatin levels in the inflorescence meristem. Moreover, *OsCKK2 RNAi* lines better tolerate salt stress showing decreased growth penalties, higher number of panicles, increased levels of photosynthetic pigments and photosynthetic rates, improved water content and decreased oxidative damage determined by electrolyte leakage [76].

At a molecular level, changes in cytokinin concentrations influence stress responses most probably by altering ROS production. Cytokinin-treated plants or mutants with altered cytokinin production or degradation exhibit ROS homeostasis imbalance, which in turn would have an effect on the activity of ROS-scavenging enzymes, on lipid peroxidation and on the expression of genes involved in photosynthesis and abiotic stress responses [56,58,69,70,77,78,79,80,81].

A detailed transcriptomic analysis of barley plants expressing *CKX1* in roots revealed attenuated cytokinin response through the HvHK3 cytokinin receptor and upregulation of two transcription factors implicated in stress responses [59]. RNA-seq analysis further suggests crosstalk with auxin as several *SAUR* and *Aux/IAA* genes exhibited lower expression in transgenic plants than in wild types, thus indicating higher auxin turnover in transgenic roots [60].

### 2.2. Stress-Induced Modulation of Auxin and Cytokinin Metabolic Routes

Extracellularly-secreted plant peroxidases, which catalyze the decomposition of peroxides, are involved in the catabolic oxidation of IAA. These oxidase-associated auxin oxidase activities impact auxin stability and level [82], as observed in UV-treated tobacco and duckweed (*Spirodela punctate* and *Lemna gibba*) plants [83]. Furthermore, IAA peroxidases are important for the control of IAA levels during root initiation and development [84] and during the process of hypocotyl elongation [85]. Abiotic stress also affects the expression of genes involved in auxin metabolism [24,86]. Auxin UDP-glucosyltransferases are strongly induced under photorespiration and H_2_O_2_-inducing conditions [87]. *Arabidopsis* UDP-glucosyltransferase UGT74E2 catalyzes the addition of glucose (glc) preferably to the auxin indole-3-butyric acid (IBA) [72]. Interestingly, ectopic overexpression of UGT74E2 in *Arabidopsis* not only increases IBA-glc concentrations, but also elevates free IBA and modifies the conjugated IAA pattern. Additionally, perturbed auxin homeostasis in transgenic plants steers architectural changes, including increased shoot branching and altered rosette shape, and it leads to enhanced drought and salt stress resistance. Moreover, IAA-glc and IBA-glc levels increase in wild-type and overexpressor plants under osmotic stress [72]. Recently, another UDP-glucosyltransferase was shown to play a role in stress response. *Arabidopsis* plants constitutively expressing the stress-regulated *UGT85U1* gene isolated from stigmas of saffron (*Crocus sativus*) exhibit enhanced salt and oxidative stress tolerance. Although auxin levels and *PIN2* expression in the roots are higher in *UGT85U1* overexpressors as compared to wild-types, there are no data regarding its targets or its implication in hormone homeostasis [88]. Stress-induced changes in the endogenous auxin pool can also be regulated through negative feedback by auxin-inducible GRETCHEN HAGEN 3 (GH3) enzymes, which catalyze amide conjugation of IAA with amino acids [89]. *GH3* gene expression has been induced in different species by various stress conditions, such as drought, salinity, cold and heat [90,91,92,93]. The rice *OsGH3-2* gene is induced by drought and suppressed by cold, and its overexpression causes significant morphological aberrations related to IAA deficiency, reduced free IAA levels, greater stomata aperture, faster water loss and hypersensitivity to drought stress [90]. Moreover, salinity stress has been shown to decrease free IAA concentration [92,94], and poplars can use IAA-amide conjugates as a source of auxin to balance the effects of salt stress on auxin content [94]. Overexpression of the *Arabidopsis GH3* gene *WES1* leads to reduced growth and increased stress tolerance by promoting the IAA-aspartate catabolic pathway [91]. In rice, activation of *TLD1*, a *GH3.13* gene, which is suppressed in aboveground tissues under normal conditions, but dramatically induced by drought stress, compensates for alterations in plant architecture and tissue patterning with an increased drought tolerance [93]. Chickpea (*Cicer arietinum*) *CaGH3-1* and *-7* and *Medicago truncatula MtGH3-7*, *-8* and *-9* were also found to be highly induced under drought and/or salt stresses, suggesting their role in abiotic stress responses [95]. In maize, the responsiveness of *ZmGH3* genes to a wide range of abiotic stresses and stress-related hormones suggests that *ZmGH3s* are auxin-stress crosstalk regulatory targets. Moreover, the expression of *ZmGH3* genes showed different patterns between shoot and root [96]. IAA-ALANINE RESISTANT 3 (IAR3), an IAA-alanine hydrolase that releases bioactive IAA from IAA-alanine, is another component of the ROS–auxin crosstalk regulatory network involved in stress adaptation. *Arabidopsis* roots sensing high osmotic stress signal the repression of *miRNA167*, which targets *IAR3* mRNA. In turn, upregulation of IAR3 [97] underpins increased local IAA levels, changes in root architecture and increased stress tolerance. In conclusion, even if IAA conjugates are considered to be either reversible or irreversible storage compounds, a direct signaling function of these conjugates in various processes including stress responses cannot be excluded. For example, *Arabidopsis* seedlings treated with IBA or IBA-glc delayed flower induction [72] and recently IAA-aspartate were suggested to have a direct and specific effect on salinity and heavy metal stress responses in pea plants [98].

Glycosylation of cytokinin also plays an important role during stress responses. Cytokinins mainly exist in the form of conjugations in plants. Reversible glycosylation of cytokinins (*O*-glycosylation and *O*-acetylation) creates as stable storage forms of the hormone. *N*-glycosylation of cytokinin is thought to be irreversible. In *Arabidopsis*, UGT76C1 and UGT76C2 catalyze *N*-glycosylation of most cytokinin species, while UGT85A1, UGT73C1, and UGT73C5 catalyze *O*-glycosylation of tZ and dihydrozeatin [18]. The *Arabidopsis* UGT76C2 catalyzes the glycosylation of all classical cytokinins and is transcriptionally downregulated by osmotic and drought stresses. Plants overexpressing *UGT76C2* are sensitive to mannitol during germination, but tolerant to drought stress as adult plants. In contrast, the knockout *ugt76c2* mutants show the opposite responses to both stresses, indicating a protective role against stress at the adult developmental stage [99]. It is known that local overproduction of endogenous cytokinin and auxin can control stress-adaptation responses by modulating ROS homeostasis [70,72]. Therefore, tissue-specific ROS levels could act as an integrator of the responses triggered by both pathways and relevant for stress-induced growth shaping. Reciprocally, direct auxin and cytokinin oxidation by spontaneous reaction with increased local ROS accumulation [100,101] may act as an alternative ROS energy dissipation pathway, albeit one minor in relevance as compared to enzyme-catalyzed degradation.

### 2.3. Modulation of Auxin and Cytokinin Transport by Stress

The hallmark of stressed plants is the accumulation of flavonoids, which seem to be negative regulators of polar auxin transport [101,102,103,104] and lead to the activation of auxin-dependent stress responses [105]. It is known that flavonoids affect the expression, localization and recycling of PINs, as well as the activity of ABCB-type auxin transporters [106,107,108,109]. Alteration of flavonoid accumulation in flavonoid-deficient mutants affects lateral root initiation and root architecture [110]. The *WRKY23* gene is induced by auxin and encodes a member of the large WRKY family, a plant-specific class of transcription factors that has been associated with responses to pathogen attack, mechanical stress and senescence [111]. WRKY23 controls proper root growth and development by stimulating the local biosynthesis of flavonols, a subgroup of flavonoids [112]. *GLUTATHIONE S-TRANSFERASE Phi 2* (*GSTF2*) transcripts are expressed in the shoot-root transition zone and root distal elongation zone of seedlings, and they respond to auxin and oxidative stress treatments. Competition between IAA and flavonoids for binding to GSTF2 may contribute to the regulation of auxin transport under normal and detrimental growth conditions [113]. By controlling the processes of auxin transport and distribution, flavonoids modulate not only plant responses to stresses, but also the development of stress induced morphogenetic responses, which alter plant growth and development in order to decrease damage of tissues or organs caused by stressors [86]. For example, peroxidase-catalyzed oxidation of IAA during UV stress is promoted by flavonoids and modulates leaf and plant architecture [83]. In opposition, auxin affects flavonoid metabolism by inducing UDP-glucose:flavonoid 3-*O*-glucosyltransferase activity [114]. An interesting recent model proposes that flavonols function as positional signals, integrating auxin, cytokinin and ROS signaling in root meristem to control root light avoidance and root growth [115]. An additional level of complexity to the possible ways by which flavonols influence auxin distribution is seen in the finding that flavonol glycosides affect auxin metabolic turnover [104].

Given that they are responsible for the asymmetric distribution of auxin, it is scarcely surprising that some components of the polar cell-to-cell auxin transport machinery are primary targets of environmental signals. Interestingly, synthetic polar auxin transport inhibitors 2,3,5-triiodobenzoic acid and 1-*N*-naphthylphthalamic acid induce morphological changes similar to those imposed by stress, including decreased root elongation, increased root hair density, decreased leaf size, inhibition of mesophyll cell expansion and fluctuations in chlorophyll content [72,116]. Temporal-spatial patterns of PIN FORMED (PIN) auxin efflux carriers and their subcellular localization cooperatively modulate stress-induced reorientation of growth. In *Arabidopsis* roots, for example, salinity treatments in 21-day-old seedlings were shown to induce expression of *PIN1*, *PIN3* and *PIN7* genes [117], while in seven-day-old seedlings, mRNA and protein levels were significantly reduced [118]. In the case of *PIN2*, five-day-old seedlings downregulated its expression in response to salinity [119], while high osmotic stress imposed by mannitol upregulated *PIN3* expression in adult plants [97]. Inter- and intra-cellular auxin transport is also mediated by AUX1/LAX (AUXIN RESISTANT1/LIKE-AUX1) influx transporters, ATP Binding Cassette (ABC) transporters and putative auxin transporters PILS (PIN-LIKES). Transcriptional analysis in maize revealed that stress responsive *ZmLAX*, *ZmPIN*, *ZmPILS* and *ZmABCB* genes have the opposite expression pattern in shoots and roots during salt, drought or cold treatments [120]. 

Dynamic subcellular PIN localization visible as a constitutive endocytic recycling [121] provides a plausible mechanism for rapid reshuffling of PIN proteins between different sides of the cell [122]. Thus, changes in PINs’ polar localization in response to different developmental or environmental signals could dynamically change the auxin flow, with a concomitant impact on physiological and morphological processes of the plant [123,124,125,126,127]. Cold stress has been shown to inhibit basipetal auxin transport by interfering with the trafficking of PIN2. Short-term cold stress application did not affect the asymmetric localization of PIN2, but suppressed cellular trafficking. Moreover, lateral relocalization of PIN3, which mediates the early phase of root gravity response, was also inhibited by cold stress in *Arabidopsis* roots [128]. It was recently shown that osmotic perturbation influences the balance between endocytosis and exocytosis in root meristem. Acute hyperosmotic stress attenuates exocytosis by boosting clathrin-mediated endocytosis, whereas the opposite effect is observed from hypo-osmotic stress treatments [129]. In addition, as an adaptive mechanism, changes in auxin redistribution in root tips triggered by salt-induced endocytosis of PIN2 allow directional bending of the root away from the stressor [130]. However, knowledge as to the molecular mechanism involved in membrane trafficking of polar-localized PINs upon stress is still limited. Therefore, to obtain a more comprehensive view on how auxin flux is adapted in response to stress, more focused studies are needed on cellular regulation of membrane traffic under stress. 

In contrast to auxin, little is known about cytokinin cell-to-cell transport, and consequently, its role in plant adaptation under abiotic stress conditions remains as yet undiscovered. The presence of cytokinin in xylem and phloem sap indicates that cytokinin can be transported over long distances acropetally and basipetally [131,132,133]. Cytokinins are translocated by the xylem via acropetal transport, as tZ-ribosides from root to shoot [132,134], and by the phloem via basipetal transport as iP-type from shoot to root [135]. Moreover, basipetal transport of cytokinin occurs through symplastic connections in the phloem and stabilizes the root vasculature pattern [136]. Two purine permeases involved in cytokinins cellular transport have been described in *Arabidopsis*: PUP1 and PUP2 [132,134]. Transport studies in cell cultures and yeast indicated that adenine and cytokinins are transported by the PUP system. Direct measurements demonstrated that PUP1 is capable of mediating uptake of radiolabeled tZ, while PUP2 is able to transport a number of cytokinins. Presence of PUP2 in the phloem suggests a function in the long-distance transport of cytokinins [134]. In addition, entry of zeatin-type cytokinins into the xylem transport stream is regulated by ABCG14. This transporter is mainly expressed in the plasma membrane of pericycle and stellar cells of roots, overlapping with the expression pattern of *IPT3* and *CYP73A2*, and it has been suggested to function as an efflux pump that plays an essential role for root to shoot translocation of the root-synthesized cytokinins [137,138]. ABCG14 could represent the major root-to-shoot transporter of cytokinin. However, the possibility that other transporters may also contribute in this process cannot be excluded. 

Therefore, deciphering how auxin and cytokinin transport and distribution are linked by stress-adaptation responses represents a novel avenue with incredible potential for understanding the morphological changes and decreased growth rates observed in plants exposed to environmental hardships.

### 2.4. Auxin and Cytokinin Signaling Circuits Influenced by Stress

A decrease in the activity of the *DR5:: GUS* auxin marker [139] during drought [41,140], salt [118] or O_3_ stress [141] indicates stress-induced auxin signaling attenuation. 

TIR1/AFB1-5 F-box nuclear auxin co-receptors [142] are involved in tolerance to stress. *TIR1, AFB1*, *AFB3* and *AFB5* expression is downregulated by oxidative stress [141]. Stress resilience in *tir1 afb2* mutants was manifested by reduced accumulation of hydrogen peroxide and superoxide anions, as well as enhanced antioxidant enzyme activities under oxidative and salt stress. Moreover, *tir1 afb2* showed increased tolerance against salinity measured as chlorophyll content, germination rate and root elongation compared to wild-type plants [143]. Eight *TIR1* homologous genes (*PtrFBLs*) in *Populus trichocarpa* differentially respond under heat stress, and *35S::PtrFBL1* plants exhibited increased susceptibility to drought stress [140].

Expression of many gene family members involved in nuclear auxin signaling, including auxin response factor (ARF) transcription factors and the early auxin-responsive genes (*Aux/IAA*, *GH3*, *SAUR* and *LBD*), is affected by abiotic hardships [141,144,145,146]. Moreover, stress modulation of auxin signaling also seems to be conserved in different plant species [141,143,144,145,146]. Comprehensive expression profiling of auxin-related genes in *Sorghum bicolor* revealed that three genes (*SbIAA1, SbGH3–13,* and *SbLBD32*) were highly induced under salt and drought treatment [146]. Furthermore, a detailed analysis of auxin signaling in *Arabidopsis* showed that transcripts of several Aux/IAA transcriptional repressors were reduced in response to O_3_-induced apoplastic ROS, with the exception of *IAA10* and *IAA28* transcripts, the levels of which were transiently increased [141]. In rice, OsIAA6 was shown to be involved in drought stress. The overexpression of *OsIAA6* under the constitutive phosphogluconate dehydrogenase (PGD) promoter (*PGD1:OsIAA6*) improves the tolerance of transgenic plants to drought. Moreover, transcript expression levels of the dehydration marker dehydration inducible protein 1 are repressed in *PGD1:OsIAA6* as compared to wild-type plants. Additionally, it seems that OsIAA6-mediated drought responses might control auxin biosynthesis, as transgenic lines exhibit higher expression of *YUC* genes [147]. Abiotic stress can also influence auxin signaling by modulating AUX/IAAs stability. In *Arabidopsis*, salt-induced nitric oxide (NO) accumulation was proposed to lower free IAA pool size in root meristem by signaling the downregulation of *PIN* genes expression. As a result, stabilization of AUXIN RESISTANT3 (AXR3)/IAA17 protein led to the inhibition of root meristem growth [118]. In sorghum, salinity strongly induces many *ARF* genes in leaves while downregulating them in roots. Only *SbARF10*, *SbARF16* and *SbARF21* genes are induced in roots by salinity [146]. In contrast, most *Arabidopsis ARF* genes are negatively regulated by drought and salt stresses [148]. *OsARF11* and *OsARF15* in rice and *GmARF33* and *GmARF50* in soybean (*Glycine max*) are among the drought-responsive gene targets [144,145]. Abiotic stress regulation of *ARF* expression could occur in part via *miRNAs*, inasmuch as, for example, salt stress-induced *miRNAs* were shown to target *ARFs* in radish (*Raphanus sativus*) [149], and induction of *Arabidopsis miRNA167* upon stress could cleave *ARF6* and *ARF8* transcripts [150]. 

In *Arabidopsis*, the cytokinin signaling cascade is a multi-step phosphorelay consisting of histidine protein kinase (AHK), histidine phosphotransfer proteins (AHPs) and response regulators (ARRs) [151]. ARRs are divided into two classes: type-A partially redundant negative regulators and type-B positive regulators [152,153,154,155]. While phosphorylation of type-A ARRs stabilizes them, phosphorylation of the type-B ARRs regulate transcription of cytokinin-activated targets, including type-A ARRs, which are strongly and rapidly induced in response to cytokinin [155,156,157,158]. These cytokinin signaling components’ respective genes are differentially affected by various stresses [48]. The expression of the *Arabidopsis* cytokinin receptors *AHK2*, *AHK3* and *AHK4* is rapidly induced by dehydration stress [159], suggesting that elevated cytokinin perception might play a role in stress response. *AHK2* expression decreases by approximately half in the first hours after transfer to low water potential while *AHK4* expression is only slightly changed [160]. Moreover, downstream components of the cytokinin signaling pathway respond to harsh environments. Transcripts of type-A ARR7 are induced by cold, drought and high salinity [117,161,162], whereas *ARR5*, *ARR6* and *ARR15* are induced by salinity and dehydration stresses [117,162,163]. Heat stress downregulates in leaves the expression of *AHK2*, *AHK3* and *AHK4* genes, as well as type-A *ARR8* and *ARR9* and type-B *ARR10* and *ARR12* response regulator genes [80]. The effect of cytokinin upon water deficiency is linked to ABA homeostasis and accumulation of the osmoprotectant proline [58,160]. Moreover, cytokinin redox control of plant development upon stress may be appreciated through its effect on the expression of stress-induced genes encoding proteins involved in secondary metabolism such as flavonoid and phenylpropanoid biosynthesis, a set of glutaredoxin, peroxidase and glutathione transferase genes and antioxidant enzyme genes [164,165,166,167]. We further will summarize here the efforts that have been made to work out the roles of the different components of the cytokinin signaling pathway on stress adaptation and response. Genetic analyses indicate that cytokinin receptors have tissue- and stress-specific function in abiotic stress response. Mutants lacking the functional cytokinin receptors are more resistant to drought, salt and cold stress [159,161,168]. The *Arabidopsis* loss-of-function *ahk2* and *ahk3* single mutants exhibit enhanced dehydration and salinity tolerance as compared to wild-type plants, and the effect is even more pronounced in the double *ahk2 ahk3* mutant [159,168]. Similarly, *ahk2 ahk3* and *ahk3 ahk4* were significantly more resistant to freezing temperatures [161]. In the aforementioned works, the stresses imposed were severe or near lethal and shoot greenness and plant stress survival were used as indicators of stress resistance. Kumar and Verslues [160], however, used controlled mild stress conditions to study the effect of stress on growth by measuring root elongation and total fresh weight in seedlings. The enhanced root elongation phenotype of *ahk3-3* mutants [169] was more pronounced at low water potential induced by polyethylene glycol, but neither increased root elongation, nor increased fresh weight were observed under chronic salt stress. However, *ahk2-2* mutants were more sensitive to moderate severity of salt stress as measured by a greater reduction in root growth and decreased fresh weight as compared to the wild-type. The authors also found a synergistic effect in the fresh weight under chronic salt stress in *ahk3 ahk4* double mutants, the weight of which was much less affected in shoots than in the cases of any of the other single or double mutants. Therefore, the data indicate a specific role for AHK2 in root growth during long-term salt stress and for AHK3 and AHK4 in response to decreased water potential in shoots [160].

Three *Arabidopsis* AHPs (AHP2, AHP3 and AHP5) control responses to drought stress in a negative and redundant manner. Loss of function of these three *AHP* genes resulted in a strong, drought-tolerant phenotype that was associated with the stimulation of protective mechanisms, such as improvement in cell membrane integrity [170]. In rice, OsAHP1 and OsAHP2 were found to act as negative regulators of the osmotic stress response. The *OsAHP RNAi* rice plants displayed strong osmotic tolerance with increased root fresh weight [171]. In addition, a positive role of ARR1, AHP2, AHP3 and AHP5 was described during cold stress and mediated by regulating the expression of type-A *ARRs*. The *arr1* mutation, as well as *ahp2 ahp3 ahp5* triple mutations greatly reduced the cold induction of *ARR5*, *ARR6*, *ARR7* and *ARR15* genes. Furthermore, it has been demonstrated that response to cold stress works downstream of AHK2 and AHK3 receptors [162]. Similarly, in response to salt stress, the activation of ARR1- and ARR12-dependent signaling pathways in roots could modulate *ARR5* induction [172].

In addition to the core components of the cytokinin signaling pathway, other downstream targets have been linked to abiotic stress response. CYTOKININ RESPONSE FACTORS (CRFs), members of the APETALA2 (AP2) family, are transcriptionally upregulated by cytokinin and regulate transcription of a large portion of cytokinin-response genes, many of which are also differentially regulated by type-B ARRs [173]. Examination of tomato *SlCRF1* and *SlCRF2* transcripts during abiotic stress revealed that the two genes have patterns of regulation distinct from one another and between shoot and root tissues. Cold highly induced expression of *SlCRF1* in leaves and roots, whereas heat repressed its expression in roots. In contrast, *SlCRF2* expression was induced in roots by oxidative stress [174]. In addition, analysis of *Arabidopsis CRF6::GUS* revealed that *CRF6* promoter activity is elevated by endogenous production of hydrogen peroxide [175], under heat shock, as well as salt and oxidative stress conditions [176]. Moreover, constitutive overexpression of *CRF6* led to improved photosynthesis and increased root growth when exposed to oxidative stress-inducing conditions [177]. *SlCRF5*, a tomato CRF6 orthologue, is also induced by heat, hydrogen peroxide and drought [178]. In addition, retrograde signaling in response to mitochondria dysfunction controls *CRF6* and *CRF5* expression [179,180]. Recently, *Arabidopsis* CRF6 was proposed as a component of the ROS–cytokinin crosstalk regulatory network, serving to attenuate cytokinin signaling as part of an adaptive response to stress. In response to hydrogen peroxide treatment, CRF6 represses the expression of genes involved in the cytokinin signaling pathway, which include *AHP1*, type-A *ARR6* and *ARR9* and type-B *ARR1*. In addition, cytokinin biosynthesis *LOG7* and cytokinin transport *ABCG14* genes are also repressed by CRF6 [177]. Examination of *Arabidopsis* CRFs showed strong transcriptional induction of *CRF4* in shoots and roots after exposure to cold. *CRF4* overexpressors and loss-of-function *crf4* plants exposed to cold stress showed no differences from untreated plants in term of stress tolerance. Upon exposure to freezing temperatures, however, a positive correlation between *CRF4* expression levels and stress tolerance was observed [181]. Finally, ROS- and redox-responsive protein modification could sharpen hormone signals during stress acclimation responses since TIR1 and AHPs are targets of NO *S*-nitrosylation [182,183].

## 3. Auxin–Cytokinin Crosstalk

Given that the physiologic effects of auxin and cytokinin depend largely on their concentrations, mechanisms regulating their synthesis and breakdown are very important for different developmental processes. Besides, interactions between auxin and cytokinins operate extensively via reciprocal influences on each other’s metabolism [184]. Evidently, crosstalk between these phytohormones also occurs via co-regulated genes and shared signaling components. In addition, the crosstalk is spatially and temporally regulated, providing thus adaptability and fine-tuning of responses. In recent years, crosstalk between the two hormones has been studied extensively at all levels: synthesis, perception and transport; and we are beginning to understand how these networks interact to control a wide variety of plant responses [3,8,19,185,186,187]. Here, we summarize current knowledge on auxin-cytokinin crosstalk pathways.

### 3.1. Metabolism-Related Auxin-Cytokinin Crosstalk Components

Auxin-mediated regulation of cytokinin synthesis was first observed in *Arabidopsis* roots treated with auxin, which led to upregulation of the *IPT5* and *IPT7* genes [188]. In the case of *IPT5*, upregulation by auxin is mediated by SHY2/IAA3 [189,190]. In shoots, in vivo deuterium incorporation experiments have shown that auxin mediates a very rapid negative control of the cytokinin pool mainly by suppressing the biosynthesis of tZ via the isopentenyladenosine-5′-monophosphate iP nucleotide-independent pathway [191]. In addition to its role in cytokinin biosynthesis, auxin has also been shown to affect cytokinin degradation by downregulating *CKX2, CKX4,* and *CKX7* gene expression and upregulating *CKX1* and *CKX6* in *Arabidopsis* auxin-treated seedlings [192]. Moreover, the *Arabidopsis CKX6* gene is rapidly and transiently induced by auxin during leaf development in canopy shade [193].

Elevated cytokinin levels lead to a rapid increase in auxin biosynthesis in young, developing root and leaf tissues, whereas decreased endogenous cytokinin levels reduce auxin biosynthesis in six days after germination in whole seedlings and root meristem tissues [194]. Analysis of auxin biosynthesis pathways revealed that in root meristems, enzymes from the tryptophan-dependent IAA biosynthesis pathway are regulated by cytokinin at transcriptional level. These activities include those of anthranilate synthase alpha subunit 1 (ASA1), anthranilate transferase 1 (PAT1), indole-3-glycerolphosphate synthase (IGPS), cytochrome P450s CYP79B2 and CYP79B3, YUC5, YUC6, YUC5-like, tryptophan aminotransferase (TAA1); as well as of enzymes from the indole-3-acetonitrile (IAN) pathway, nitrilase 1 (NIT1) and nitrilase 3 (NIT3), which catalyze the conversion of indole-3-acetonitrile (IAN) to IAA. In addition, auxin conjugation was also found to be affected by cytokinin via regulation of *GH3.17* and *GH3.9* expression [194].

Reduction of IAA levels in *Arabidopsis* aerial tissue was observed in six-leaf stage *35S::CKX1* and *35S::CKX2* seedlings [61]. On the other hand, expression of *AtCKX3* in tobacco plants does not affect IAA content in leaves [195]. Therefore, contradictions regarding the reciprocal interplay between cytokinin and auxin rely on the fact that auxin-cytokinin crosstalk pathways are developmental stage dependent and tissue specific. For example, cytokinin and auxin interactions in *OsCKX2 RNAi* plants are observed through the reduction of IAA levels triggered by accumulation of tZ-type cytokinins in the panicle region. The increased number of spikelets and hence grain yield in mutant plants indicates enhancement of inflorescence meristem activity due to a more favorable cytokinin-auxin ratio in this secondary meristem [76].

### 3.2. Signaling-Related Auxin-Cytokinin Crosstalk Components

The interaction of auxin and cytokinin signaling pathways is crucial in the control of shoot apical meristem activity and specification of embryonic roots. It has been shown that the negative regulators of the CK signaling pathway, the type-A ARR7 and ARR15, integrate cytokinin and auxin signals in the embryonic root, as well as in shoot-stem cell niche [196,197]. Auxin-controlled ARR7 and ARR15 activity is necessary for proper embryo development, and *Arabidopsis* embryos in which neither of the genes are functional show strong patterning defects [196]. In the shoot meristem, *ARR7* and *ARR15* expression is induced by cytokinin, whereas auxin has a negative effect. This is, at least in part, mediated by the AUXIN RESPONSE FACTOR5/MONOPTEROS (MP) transcription factor. These regulatory mechanisms confirm antagonism between auxin and cytokinin in the root meristem, even as they suggest a synergy between the two hormones in the shoot apical meristem, and this is well supported by classic shoot regeneration experiments [197]. In root apical meristems, interaction between auxin and cytokinin signaling pathways was also found to be mediated through Aux/IAA SHORT HYPOCOTYL2 (SHY2), a repressor of auxin signaling [189]. Thereby, ARR1 and ARR12 activate *SHY2* transcription specifically at the vascular tissue of the transition zone of root meristem, which negatively regulates *PIN1*, *PIN3* and *PIN7* expression. Subsequent changes in auxin levels promote cell differentiation and lead to decrease in root apical meristem size [189,198]. Additionally, auxin-dependent degradation of SHY2 is required for the induction of *ITP5* expression at the transition zone, as IPT5 activity is lost in the *shy2-2* mutant [189]. Accordingly, it has been shown that cytokinin reduces auxin efflux from cultured tobacco cells [199], and it negatively regulates the expression of most of the *PIN* genes in roots [189,199,200]. An exception is seen in *PIN7* expression, which is induced by cytokinin in *Arabidopsis* roots [136,200,201]. Besides a transcriptional regulation, cytokinin was found to regulate endocytic recycling of the auxin efflux carrier PIN1 by redirecting it for lytic degradation in vacuoles. Stimulation of the lytic PIN1 degradation is not a default effect for the general downregulation of proteins from plasma membranes, but rather it is a specific mechanism to rapidly modulate the auxin distribution in cytokinin-mediated developmental processes [200]. During lateral root initiation, for example, AHP6-dependant inhibition of cytokinin signaling allows the correct PIN1 localization and thus the formation of the auxin gradient required to pattern lateral root primordia [202]. 

Some cases of hormonal interactions to distinguish between primary, secondary or tertiary regulation are still not well understood [203]. Auxin-cytokinin crosstalk is spatially and temporally regulated, and interactions with other hormones must also be considered in order to understand developmental processes and responses to the environment fully. Further studies using genome-wide profiling for epigenetics, transcriptomics and proteomics will provide new information about the interconnected web of hormone actions.

## 4. Abiotic Stress-Auxin-Cytokinin Transcriptional Crosstalk Networks

**Figure 1:**
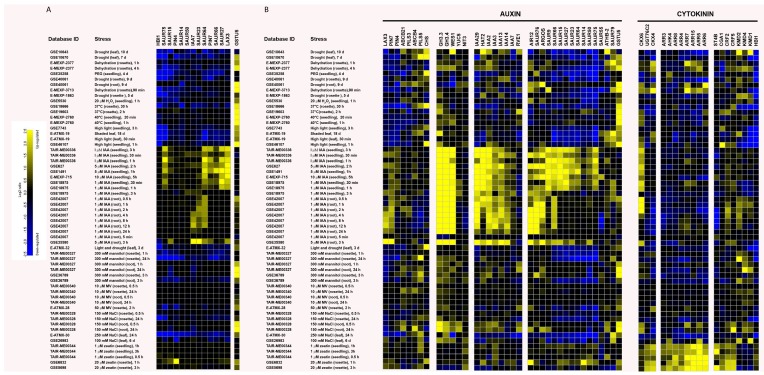
Auxin- and cytokinin-related genes differentially expressed by both hormones and various environmental cues. From 335 auxin- and 128 cytokinin-related genes selected from the literature and The Arabidopsis Information Resource (TAIR, Available online: www.arabidopsis.org), probes for 246 (auxin) and 128 (cytokinin) genes were found using the Genevestigator database and Affymetrix Arabidopsis ATH1 Genome Array (Available online: http://www.genevestigator.com/gv/index.jsp). We analyzed their expression upon 59 perturbations from 328 microarrays. Using perturbation tool analysis and applying fold change cut-offs of >1.5 and significance *p*-values of <0.05, we ended up with a total of 36 auxin-related genes and 19 cytokinin-related genes that responded to both hormones and to single or multiple stressors at the same time. The figure shows the selected experiments, which can be retrieved using the unique database ID for each. The genes’ expression responses are calculated as log_2_-ratios between the signal intensities from different perturbations as compared with control or mock-treated samples. (**A**) The panel shows a hierarchical clustering heat map from genes downregulated or upregulated simultaneously by all selected stresses. The hierarchical clustering maps for the total number of selected genes. (**B**) The panel shows the expression profile of auxin-related genes divided into 5 groups according to their roles (transport, metabolism, biosynthesis, signaling, and response genes) and 3 groups for cytokinin-related genes (metabolism, perception and signaling, and response-related genes). Gene expression patterns are represented in a log2 ratio ranging from −2.5 (blue color, down-regulated) to +2.5 (yellow color, up-regulated). Abbreviations: d = days; min = minutes; h = hours; PEG = polyethylene glycol; MV = methyl viologen.

As occurs with crops in the field, it is of pivotal importance to understand at the cellular and molecular levels how plant growth is adapted to a combination of environmental cues in order to design specifically tailored biotechnology tools. Previously, transcriptome analysis of the response of the complete set of genes involved in cytokinin signaling and metabolism to different environmental hardships showed that *IPT3*, *IPT5*, *CYP735A2*, *LOG5*, *CKX4*, *ARR10* and *CRF6* are the most responsive genes [204]. Here, we analyzed the response of the complete set of auxin and cytokinin pathway genes to different abiotic stress conditions, as well as to cytokinin and auxin treatments. To perform the analysis, we used gene expression data from *Arabidopsis* ecotypes Col-0, which are publicly available in the Genevestigator database [205]. We considered various kinds of abiotic stress: oxidative stress induced by exposure to high light (HL), heat, H_2_O_2,_ or the ROS propagator methyl viologen (MV); osmotic stress imposed by exogenous addition of mannitol, polyethylene glycol or NaCl; dehydration and drought. Auxin- and cytokinin-related genes simultaneously affected by both hormones and stressors were selected. It is also possible to compare their expression in different tissues such as roots, leaves and seedlings depending on the array experiments (Figure 1). These target genes could represent important auxin and cytokinin interaction hubs to control the dynamic behavior of cellular processes related to stress-induced reorientation of growth and thus relevant genetic tools to provide farmers with climate-resilient crops with improved yield and which mitigate unwanted morphological traits associated with stress.

In Figure 1A, genes displaying similar transcriptional response upon oxidative and osmotic stresses were grouped by hierarchical clustering analysis. Cluster 1 group genes downregulated by stress and induced by auxin and cytokinin treatments include the cytokinin-responsive basic helix-loop-helix *HBI1*, *SAUR14*, *15*, *23* and *50*, and auxin transporter *PIN4*. In addition, genes not responsive to H_2_O_2_ or methyl viologen (MV) treatments belonging to this cluster are the auxin response regulator *IAA7*, auxin transporters *PIN7* and *LAX3* and *SAUR27*, *64*, *66* and *75*. Cluster 2 is composed only of the auxin-inducible and glutathione (GSH)-dependent GLUTATHIONE S-TRANSFERASE TAU 5 (GSTU5) encoding gene, which is upregulated by all selected stressors. We hypothesize that these genes represent general components of the environmental stress response playing a crucial role in plant phenotype plasticity. In other words, they could constitute central components of the auxin-cytokinin regulatory transcriptional network, functioning as regulatory hubs between growth and stress-tolerance pathways. Supporting this hypothesis, for example, is the knowledge that PIN4- and PIN7-driven auxin transport is important for shaping shoot system architecture [206]. In *Arabidopsis thaliana*, *Medicago truncatula* and *Lotus japonicas*, active transport of auxin inside the cells carried out by LAX3 is important for lateral root emergence and the arrangement of leaves on a plant stem [207,208,209]. The contribution to auxin transport mediated by MtLAX3 may also play an important role during the processes of indirect somatic embryogenesis and symbiotic nodulation, and in both *Medicago* and *Lotus*, overexpression of *LAX3* leads to increased leaf size, increased shoot branching, higher seed number and greater root nodulation [208]. SAURS are implicated in a wide range of developmental processes [210], and some from our analysis promote auxin-mediated control of plant cell elongation, including SAUR14, 15, 64, 66 and 75 [211], or cell expansion, as in the case of SAUR13 [212]. Downregulation of *IAA7* in roots could be part of a stress-adaptive response, as repression of *IAA7* in wheat roots by the transcription factor TaNAC69-1 increased root growth in drying soil [213]. Moreover, it is likely that HBI1 is a component of the central growth regulation circuit balancing growth and biotic stress responses [214].

Induction of *GSTU5* gene expression by abiotic stresses is not surprising given its peroxidase activity. Accordingly, overexpression of tau-class GST with high glutathione peroxidase activity increases chilling, osmotic dehydration, salinity and herbicide tolerance in transgenic plants [215,216,217,218,219]. As the major non-protein thiol source, GSH precisely regulates cellular redox state in association with the ascorbate system, which in turn determines growth and developmental patterns in plants by modulating processes, such as mitosis, cell elongation, senescence, cell death and stress responses [28,220,221,222]. GSH redox state controlled by glutathione peroxidases, for example, plays an important role in root architecture and lateral root development [223]. Therefore, GST activities could be among the components linking auxin and redox signaling pathways through modulation of the cellular GSH redox buffer. The direct effect on growth and development of all these target genes is in line with the structural changes in plant tissues essential for adaptation to stress-prone environments.

On the other hand, genes showing stress- and tissue-specific regulation might represent fine-tuning components that assist or contribute to the necessary readjustment of auxin and cytokinin responses leading to altered growth and development in response to signals arising from specific environmental changes. A hierarchical clustering heat map of selected general and fine-tuning genes organized according to their role on auxin or cytokinin pathways is presented in Figure 1B. Fine-tuning genes for auxin transport include *ABCB4*, *ABCB21*, *PILS3* and *PILS5*. The dual role of ABCB transporters in polar auxin transport and stress tolerance [224] could be linked to their plasma membrane localization and their facultative auxin importers/exporters activity depending on auxin cytosolic levels. Moreover, the specificity of their actions depends on their tissue-specific expression patterns. *ABCB21* is highly expressed in junctions of lateral organs within the aerial part of plants and in pericycle cells of root tips [225]. Complementarily, *ABCB4* expression is restricted to root epidermis and cap [226]. The opposite expression pattern of *PILS3* and *PILS5* genes in drought-stressed leaves points to specific roles for their respective encoding proteins in intracellular auxin homeostasis. PILS5 reduces nuclear auxin signaling and stimulates auxin conjugation [227]. Hence, intracellular compartmentalization of IAA and IAA conjugates appears to contribute importantly to stress-dependent growth regulation. Regarding the auxin biosynthesis pathway, we selected *YUC5* and *NIT3* transcripts. Interestingly, both transcripts are induced by osmotic stressors, including mannitol and drought, thereby implicating both auxin biosynthesis genes in response to low water potential. NITs play an important role in glucosinolate metabolism and camalexin homeostasis, but their function in auxin biosynthesis is in doubt [228,229]. Disruption of the glucosinolate pathway leads to auxin-overproduction phenotypes, presumably due to diversion of intermediates to IAA synthesis via NITs [229]. Therefore, NITs’ activities could indirectly regulate auxin levels under stress conditions by affecting the distribution of common auxin intermediates between YUC and glucosinolate pathways.

*GH3.3*, *GH3.4* and *WES1* transcripts, responsible for auxin homeostasis, are differentially expressed upon heat, mannitol, salt and drought treatments. GH3-mediated auxin homeostasis was proposed as a protective mechanism in response to excess of auxin [230]. In this sense, WES1 overexpression leads to smaller plants with severely dwarfed phenotype, which are more tolerant to cold, heat and drought [91].

Auxin signaling genes include a homeobox-leucine zipper gene *HAT2* that is induced by auxin plus five *IAAs*. HAT2 plays opposing roles in roots and shoots in regulating auxin-mediated morphogenesis [231]. According to the expression pattern obtained for *HAT2*, it could play a role in growth modulation in the presence of stressors affecting water potential. 

Auxin response-related genes differentially expressed upon stresses and both hormones include four *SAURs* (*9*, *55*, *76*, *79*), *AUXIN-INDUCED IN ROOT CULTURES 12* (*AIR12*), *AUXIN-REGULATED GENE INVOLVED IN ORGAN SIZE* (*ARGOS*), myb family transcription factor *REVEILLE1* (*RVE1*) and *ARABIDOPSIS THALIANA HOMEOBOX PROTEIN 2* (*ATHB-2*) genes. In accordance with the stress-specific regulation of the cell cycle [229], transcription of the positive regulator of cell proliferation *ARGOS* [232] is induced in roots under salt, mannitol or drought stress and in high light-stressed leaves, and it is repressed by heat stress in shoots. Supporting the stress adaptation role, constitutive overexpression of *ARGOS* genes leads to improved drought performance and also affects ethylene signaling in both *Arabidopsis* and maize plants [233]. The clock regulated transcription factor RVE1 promotes plant growth by regulating *YUC8* gene expression during the day [234]. Therefore, RVE1 could work as a node that connects auxin and circadian signaling networks with the external environment to control plant growth accordingly.

Among cytokinin-related genes, transcript levels of *CKX4* are particularly responsive to the environment, and CKX4 has been pointed out as one of the components of the cytokinin-stress regulatory network involved in root system architecture [204]. In line with the observed positive effect of reduced cytokinin levels in roots on plant growth and development upon stress [57], *CKX6* transcripts are upregulated upon salt, mannitol and drought treatments in roots. High light also induced *CKX6* expression, indicating that tissue specific compartmentalization of cytokinin degradation, as well as substrate preference of CKX isoforms define cytokinin signaling and stress tolerance. A regulator of cytokinin homeostasis, UGT76C2, is also present in our analysis. Its participation during heat and high light adaptation responses could be inferred through its transcriptional response, as already observed during drought and osmotic stress responses [99]. AHK2, AHK3 and AHK4 are considered to act as negative regulators of salt, drought [159,168], rapid dehydration [168] and freeze tolerance [161]. However, only *AHK4* responded against both hormones and abiotic stress (mannitol, high light and drought stresses). Therefore, it is reasonable to think that AHKs hand down specific effects depending on the type of stress and their tissue-specific expression pattern. That is in addition to the nature of the downstream AHPs and ARRs signaling pathway components that they regulate. Several type-A ARRs respond to osmotic stress, including drought, salinity and mannitol treatments. Interestingly, drought induction of *ARR5* and *ARR15* is independent of the cytokinin receptors [168], implying stress-specific regulation of their transcription. 

Cytokinin response factors present in the analysis include CRF5, CRF2, CYTOKININ-RESPONSIVE GATA TRANSCRIPTION FACTOR1 (CGA1), KISS ME DEADLY (KMD) 1, 2 and 4 and the flavonoid glycoside 7-sulfotransferase (ST4B). The F-box proteins KMD1, KMD2 and KMD4 are negative regulators of the cytokinin-signaling cascade [235] and of antioxidant phenylpropanoid biosynthesis [236], suggesting their role as coordinators of both cytokinin and stress defense systems. 

Even though the analysis has been made (1) using a pre-selection of well-characterized components of both hormonal routes and (2) only at the transcriptional level, its results augment the information about common target genes modulated by hormonal and stress circuits. Thereby, it can contribute to our understanding as to the molecular basis of plant phenotype flexibility in a changing climate.

## 5. Conclusions

In recent decades, the scientific community has independently accumulated an unprecedented amount of knowledge relating to the molecular and genetic mechanisms controlling the defense mechanisms plants use to survive sudden changes in their habitats and the physiological and developmental processes directed by phytohormones. Nevertheless, it is only in recent years that the importance of integrating the two processes has become evident, inasmuch as, in addition to interacting with one another, stress and phytohormone modules also share common components. These components belong to complex signaling networks that prevent or attenuate cellular damage upon stress by affecting plant growth and development. As a result, plants accordingly adapt their morphology to their new imposed habitat.

In this review, we aimed to provide an overview of the molecular targets that balance auxin and cytokinin homeostasis and interact with signals triggered by plant cells under unfavorable growth conditions. The increasing amount of molecular data contributes to broadening our knowledge on auxin-cytokinin crosstalk in its developmental aspects and, at the same time, introduces an additional level of complexity. In this sense, additional crosstalk mechanisms of both with other plant hormones in stress-induced growth regulation contribute to the large pleiotropy in auxin and cytokinin actions.

Future research employing computational tools for the integration of genome-scale mathematical modeling in systems biology, field and laboratory growth and development experiments and large-scale mutational analyses are pivotal to forming a deeper understanding of plant growth and development in response to environmental cues. 

Thus, the intra- and inter-cellular spatial and temporal distribution of ROS and phytohormones among plant tissues and organs could represent an attractive manipulation platform for boosting crop yields.

## Supplementary Materials

Supplementary materials can be found at www.mdpi.com/1422-0067/18/7/1427/s1.
